# Target DNA-induced filament formation and nuclease activation of SPARDA complex

**DOI:** 10.1038/s41422-025-01100-z

**Published:** 2025-03-24

**Authors:** Feng Wang, Haijiang Xu, Chendi Zhang, Jialin Xue, Zhuang Li

**Affiliations:** State Key Laboratory of Biocatalysis and Enzyme Engineering, School of Life Sciences, Hubei University, Wuhan, Hubei China

**Keywords:** Cryoelectron microscopy, Molecular biology

## Abstract

The short Argonaute-based bacterial defense system, SPARDA (Short Prokaryotic Argonaute and DNase/RNase-APAZ), utilizes guide RNA to target invading complementary DNA and exhibits collateral nuclease activity, leading to cell death or dormancy. However, its detailed mechanisms remain poorly understood. In this study, we investigated the SPARDA system from *Novosphingopyxis baekryungensis* (*Nba*SPARDA) and discovered an unexpected filament configuration upon target DNA binding, which strongly correlated with collateral nuclease activity. Filament formation and nuclease activation require a guide–target heteroduplex of sufficient length with perfect complementarity at the central region. A series of cryo-EM structures of *Nba*SPARDA complexes, loaded with guide RNA, target DNA of varying lengths, and substrate ssDNA, were determined at ~3.0 Å resolution. Structural analyses indicated that guide RNA binding induces dimerization of the *Nba*SPARDA complex, while target DNA engagement disrupts this dimerization. Further propagation of the guide–target heteroduplex triggers filament formation through a checkpoint mechanism. The *Nba*SPARDA filament consists of a backbone formed by interlocking short Argonaute proteins, with an inner layer composed of DREN nuclease domains. Filament formation leads to tetramerization of the monomeric DREN nuclease domain, activating its collateral nuclease activity against environmental nucleic acids — a feature leveraged for molecular diagnostics. For bacteria heterologously expressing the *Nba*SPARDA system, defense against invading bacteriophages and plasmids relies on filament formation. Collectively, these findings illustrate the detailed working mechanism of the *Nba*SPARDA complex and highlight the importance of its filament formation in host defense.

## Introduction

Argonaute (Ago) proteins are present across all domains of life and are classified into eukaryotic Argonautes (eAgos) and prokaryotic Argonautes (pAgos).^1,2^ eAgos control a wide range of RNA-related cellular processes, whereas pAgos are primarily involved in host defense,^3–7^ and can be further classified into long pAgos and short pAgos.^8^ Long pAgos structurally resemble eAgos, consisting of N, PAZ (PIWI-Argonaute-Zwille), MID (Middle), and PIWI (P-element induced wimpy) domains.^1,9^ Short pAgo proteins contain only the MID domain and a catalytically inactive PIWI domain. However, they often genetically and physically associate with effector proteins that contain a conserved APAZ (analog of PAZ) domain and divergent enzymatic domains, which compensate for PIWI inactivity and confer host immunity.^8,10^

Accounting for ~60% of pAgos, short pAgo systems are widespread in prokaryotic genomes, and APAZ-containing effector proteins show significant diversity in the composition of enzymatic domains.^8,10,11^ These enzymatic domains include the NADase domain TIR (Toll-interleukin-1 receptor) from the SPARTA (Short Prokaryotic Argonaute TIR-APAZ) system,^10,12–18^ the NADase domain SIR2 (sirtuin domain 2) from the SPARSA (Short Prokaryotic Argonaute SIR2-APAZ) system,^19–21^ and nuclease domains such as DREN (DNA and RNA effector nuclease, also called DUF4365), SMEK, MRR, and HNH from the SPARDA (Short Prokaryotic Argonaute and DNase/RNase-APAZ) system.^22,23^ For SPARTA and SPARSA systems, target DNA (tDNA) binding activates the NADase domains, leading to hydrolysis of cellular nicotinamide adenine dinucleotide (NAD^+^) and cell death to block plasmid transformation and viral infection.^10,24,25^ Another overlapping classification criterion based on phylogenetic analysis categorizes short pAgos into four subclades, including S1A (SIR2-APAZ-pAgo fusions), S1B (operons with SIR2-APAZ and pAgo), S2A (operons with (Mrr-)TIR-APAZ and pAgo), and S2B (operons mainly consisting of pAgo and DUF4365-APAZ or DHS-like-APAZ).^10^

Besides the effector roles played by the enzymatic domains, the structural roles of short pAgos are played by APAZ, MID and PIWI domains.^26^ APAZ domain structurally resembles the N-L1-L2 domains of long pAgos rather than the PAZ domain of long pAgos, and typically interacts with the short pAgo proteins.^27^ The MID domain of short pAgos plays the same role as that of long pAgos in interacting with guide nucleic acids, albeit with diverse preferences.^26^ MID domains, categorized into YK-type, HK-type, RK-type, and MID-OH-type, determine whether DNA or RNA can be utilized as the guide, which sequence is preferred, and whether 5′-phosphorylation or 5′-hydroxylation is preferred.^8^ Similar to that of long pAgos, the PIWI domain of short pAgos extensively interacts with the guide–target duplex, but lacks the catalytic tetrad for target cleavage.^1,9^

The SPARDA system remains less explored compared to the SPARTA and SPARSA systems. Initial study on short Ago from *Kordia jejudonensis* (*Kj*Ago) showed that it utilizes guide RNA (gRNA) to target complementary DNA, and forms a heterodimer with SMEK-APAZ protein to exhibit weak collateral nuclease activity against dsDNA and ssDNA.^22^ Similarly, the short Ago from *Thermocrispum municipal* (*Tmu*Ago) forms a heterodimer with DREN-APAZ protein to nonspecifically cleave DNA upon RNA-guided target recognition.^23^ Recent investigation of the short pAgo from *Novosphingopyxis baekryungensis* (*Nba*Ago) found that it utilizes a small gRNA with a 5′-AU preference to target ssDNA, and associates with the DREN-APAZ protein to form an *Nba*SPARDA complex to exhibit collateral nuclease activity.^28^ Activation of SPARDA by plasmids or phages leads to cellular DNA degradation resulting in cell death or dormancy. The collateral nuclease activity of DREN, a nuclease domain with conserved PD-(D/E)-XK fold that cleaves various substrates, makes it a promising tool for nucleic acid detection.^29^ SPARDA offers several advantages over Cas12 and Cas13, including a robust collateral nuclease activity, high sensitivity, and small molecular weight.^28^ Nevertheless, the biochemical details and activation mechanism of the *Nba*SPARDA system remain largely unknown. In this study, we obtained a series of cryo-EM structures of the *Nba*SPARDA complexes in the apo state, bound to gRNA, bound to tDNAs of varying lengths, and, finally, in the presence of substrate ssDNA. Our findings reveal that tDNA engagement induces filament formation of *Nba*SPARDA complex. Specifically, filament formation induces tetramerization of the DREN nuclease domain to exhibit collateral nuclease activity, eventually leading to bacterial death.

## Results

### Biochemical and structural characterization of *Nba*SPARDA complex

**Fig. 1:**
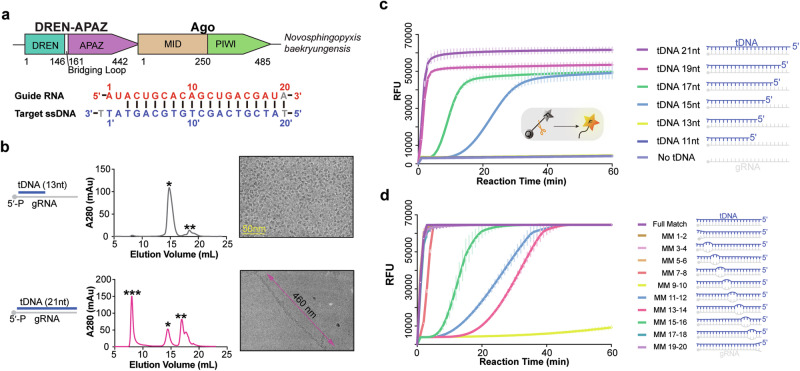
Biochemical characterization of *Nba*SPARDA complex. **a** Genome organization of the *Nba*SPARDA system, with the domain organization of *Nba*SPARDA complex color coded. **b** Size exclusion chromatography profile and cryo-EM images of gRNA-bound *Nba*SPARDA complex with tDNA of different lengths (upper: 13 nt; lower: 21 nt). The cryo-EM images are labeled with a scale bar or the length indicator. **c** FQ assay to determine the collateral nuclease activity of *Nba*SPARDA against ssDNA in the presence of gRNA and tDNA of varying lengths. **d** FQ assay to determine the collateral nuclease activity of *Nba*SPARDA against ssDNA in the presence of gRNA and tDNA containing 2-nt mismatches. FQ assay was performed in triplicate, and the error bars represent the standard deviations.

Previous study indicated that *Nba*SPARDA complex utilizes gRNA to recognize complementary tDNA, and that tDNA longer than 20 nt activates its collateral nuclease activity.^28^ In this study, His-tagged *Nba*Ago was co-expressed with strep-tagged DREN-APAZ to reconstitute the *Nba*SPARDA complex for more detailed biochemical and structural analyses (Fig. 1a; Supplementary information, Fig. S1a). To investigate the effect of tDNA length on the collateral nuclease activity, we incubated the *Nba*SPARDA complex with 5′-phosphorylated gRNA with a 5′-AU motif, and tDNA of varying lengths (11-, 13-, 15-, 17-, 19-, and 21-nt). The results showed that the collateral nuclease activity against ssDNA, measured by a fluorophore quencher (FQ) assay, is correlated with tDNA length, with 19- and 21-nt showing full activity, 17- and 15-nt showing weaker activity, and 13- and 11-nt showing no activity (Fig. 1c). The reaction mixture was subjected to either cryo-EM or size exclusion chromatography (SEC) analysis. The SEC profile indicated that 19- and 21-nt tDNA caused aggregation of most of the *Nba*SPARDA complex with a measured molecular weight of 3.46 MDa, while 17- and 15-nt tDNA caused aggregation of a small percentage, and 13- and 11-nt tDNA caused no aggregation (Fig. 1b; Supplementary information, Fig. S1b, c). The cryo-EM images indicated that the complex with 21-nt tDNA is mostly filamentous, while the complex with 13-nt tDNA is exclusively monodisperse (Fig. 1b). Moreover, introduction of mismatch at the central region (9–14) substantially compromised the collateral nuclease activity of *Nba*SPARDA and its filament formation (Fig. 1d; Supplementary information, Fig. S1d). Collectively, these observations established a strong correlation between filament formation and collateral nuclease activity, and suggested that filament formation and nuclease activation require the assembly of a guide–target heteroduplex with sufficient length and perfect match at the central region.

**Fig. 2:**
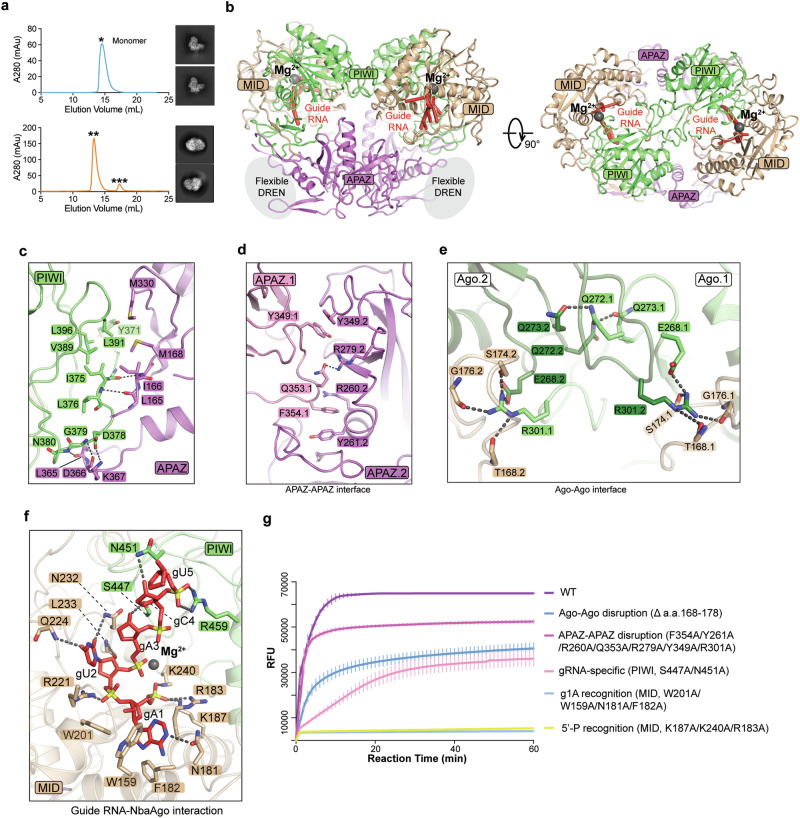
Structure of guide-bound *Nba*SPARDA complex. **a** Size exclusion chromatography profile and 2D average images of apo and guide-bound *Nba*SPARDA complexes (upper: apo; lower: guide-bound). **b** Overall structure of guide-bound *Nba*SPARDA complex from two views. **c** Interaction details between APAZ-DREN protein and *Nba*Ago protein within the *Nba*SPARDA complex. **d** Interaction details at the APAZ–APAZ interface. **e** Interaction details at the Ago–Ago interface. **f** Interaction details between gRNA and *Nba*Ago domain. **g** FQ assay to determine the collateral nuclease activity of wild-type and mutant *Nba*SPARDA against ssDNA. FQ assay was performed in triplicate, and the error bars represent the standard deviations.

To investigate the underlying working mechanism, we performed single-particle cryo-EM analysis on the *Nba*SPARDA complexes alone (termed apo complex) or loaded with gRNA (termed guide-bound complex), gRNA and a 13-nt tDNA (termed inactive complex), gRNA and a 21-nt tDNA (termed active complex), or gRNA along with a 21-nt tDNA and a substrate ssDNA (termed substrate-bound complex) (Supplementary information, Table S1). The structure of apo complex was determined at low resolution, but the 2D class averages clearly showed that it is monomeric (Fig. 2a). The structure of the guide-bound complex, determined at 2.93 Å, showed that it is dimeric with the DREN domains being flexible (Supplementary information, Fig. S2). The structure of the inactive complex, determined at 3.34 Å, showed that it is monomeric with the DREN domain being flexible (Supplementary information, Fig. S3). The structures of the active complex (Supplementary information, Fig. S4) and substrate-bound complex (Supplementary information, Fig. S5) were determined at 3.18 Å and 3.19 Å, respectively. Collectively, the *Nba*SPARDA complex undergoes a “monomer-dimer-monomer-filament” transition during the process of activation.

Based on these structures, *Nba*Ago is divided into a MID domain (amino acid (aa) 12–50) and a PIWI domain (aa 250–485), while DREN-APAZ is divided into a DREN domain (aa 1–146), a bridging loop (aa 146–161), and an APAZ domain (aa 161–442) (Fig. 1a). Extensive hydrophobic and main-chain polar contacts form between *Nba*Ago and DREN-APAZ, resulting in the formation of a tight heterodimer with a buried surface area of 4089.86 Å^2^ (Fig. 2c).

### gRNA-induced dimerization of *Nba*SPARDA complex

The guide-bound complex is predominantly dimerized, with the DREN domain missing from the cryo-EM density (Fig. 2a, b). The interactions responsible for *Nba*SPARDA dimerization occur at the APAZ–APAZ and Ago–Ago interfaces (Fig. 2b, d, e). At the APAZ–APAZ interface, F354.1 is sandwiched by Y261.2 and R260.2 to form π stacking interactions, while Q353.1 forms polar contact with R279.2, and Y349.1 forms π–π stacking interactions with Y349.2 (Fig. 2d). At the Ago–Ago interface, R301.1 of *Nba*Ago.1 inserts into a polar pocket formed by a flexible loop (aa 168–178) of *Nba*Ago.2, and forms extensive contacts with the surrounding main chains and side chains of E268.2 and S174.2 (Fig. 2e). Group alanine substitution of the interacting residues at the APAZ–APAZ interface or deletion of the flexible loop at the Ago–Ago interface disrupted the dimerization and mildly impaired the collateral nuclease activity, suggesting that the guide-induced dimerization plays a role in the process of SPARDA activation (Fig. 2g; Supplementary information, Fig. S6a).

The gRNA in the guide-bound complex is mostly flexible, except for the first five nucleotides, A1 to C5 (Fig. 2b). Extensive contacts were observed between the MID domain and the gRNA (Fig. 2f). Specifically, the 5′-phosphate group of gRNA and the phosphate group of A3 are coordinated via a magnesium ion, which facilitates the charged interactions between the 5′-phosphate groups and the side chains of K187, K240, and R183 (Fig. 2f). The bulky nucleobase of A1 inserts into a hydrophobic pocket formed by W201, W159, and F182 and forms polar contact with the side chain of N181. The size fit of this pocket and the polar contact may explain why adenosine is favored at the first position of the gRNA (Fig. 2f). The C4 oxygen of the U2 nucleobase forms a hydrogen bond with the carbonyl group of Q224 backbone, while the C2 oxygen forms a hydrogen bond with the carbonyl group in the side chain of N232, explaining why uridine is preferred at the second position of the gRNA (Fig. 2f). The main chain of L233 forms polar contact with the 2′-OH of the U2 sugar, while the side chain of S447 forms polar contact with the 2′-OH of the A3 sugar, and the side chain of N451 forms polar contact with the 2′-OH of the C4 sugar (Fig. 2f). These interactions mediated by 2′-OH explain why *Nba*SPARDA utilizes ssRNA rather than ssDNA as the guide (Fig. 2f). In addition, the side chains of R221 and R459 interact with the phosphate groups of U2 and C5, respectively (Fig. 2f).

### tDNA-induced dimer disruption and filament formation

**Fig. 3:**
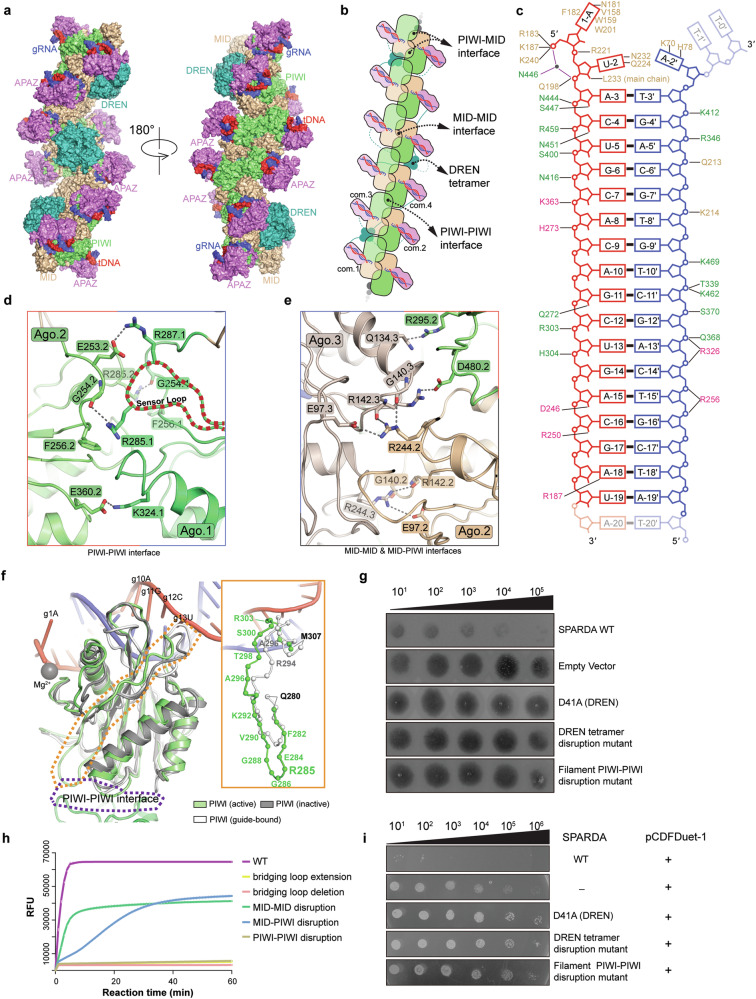
Analysis of the filament structure of the active *Nba*SPARDA complex. **a** Overall structure of the active *Nba*SPARDA complex from two different views. **b** Topological structure of the filament in cartoon representation. Com.1, com.2, com.3, and com.4 represent the first, second, third, and fourth *Nba*SPARDA complex. PIWI–PIWI, MID–PIWI, and MID–MID interfaces were highlighted with arrows and labels. **c** Interaction details between the guide–target heteroduplex and *Nba*SPARDA complex. **d** Interaction details at the PIWI–PIWI interface. **e** Interaction details on the MID–PIWI and MID–MID interfaces. **f** Superposition of the structure of inactive *Nba*SPARDA complex onto one filament structure unit of the active complex. The key structural difference was shown alongside with zoomed-in view. **g** Plaque assay to evaluate the effect of key residue mutations on filament formation and *E. coli*’s defense against T5 bacteriophage invasion. DREN tetramer disruption mutant: N17A/E13A/R20A/R33A/Q29A/E45A/D31A; PIWI–PIWI disruption mutant: R287A/E253A/K324A/E360A/R285A/F256A. **h** FQ assay to determine the collateral nuclease activity of wild-type *Nba*SPARDA or mutants against ssDNA. FQ assay was performed in triplicate, and the error bars represent the standard deviations. Bridging loop extension: GGGGG linker inserted after aa 161; bridging loop deletion: aa 146–161 deletion; MID–MID disruption mutant: R244A/G140A/R142A/E97A; MID–PIWI disruption mutant: Q134A/R295A/R142A/D480A. **i** Plasmid interference assay to evaluate the effect of key residue mutations on plasmid transformation.

The observation that the inactive complex (with 13-nt tDNA) is monomeric and the active complex (with 21-nt tDNA) is filamentous indicates that the tDNA loading would first disrupt the dimerization, and then induce the filament formation during the propagation of guide–target heteroduplex (Fig. 3a; Supplementary information, Figs. S3, S4). To figure out how 13-nt tDNA disrupts the dimerization, two copies of the inactive complex structure were superimposed onto the structure of the guide-bound complex, revealing a severe steric clash between the two duplexes, as the dimer configuration could only accommodate duplex shorter than 8 bp (Supplementary information, Fig. S6b). The mechanism by which tDNA disrupts *Nba*SPARDA dimerization is similar to that in DdmDE, a short pAgo-related host defense system.^30–32^ As shown in the representative cryo-EM images, the average length of the active complex filament is 91.61 nm, containing ~32 copies of the *Nba*SPARDA–guide–target complex (Supplementary information, Fig. S4a). The filament is composed of a backbone and an inner layer. The backbone is constituted by *Nba*Ago proteins, with the APAZ domains and guide–target heteroduplexes positioned at the periphery of the backbone (Fig. 3a). This organization tethers the DREN domains together, forming tetramers in the inner layer (Fig. 3a).

The filamentous backbone is formed in an open-ended domain swapping manner, and the intermolecular interactions occur at the MID–MID, PIWI–PIWI, and MID–PIWI interfaces in an asymmetric manner (Fig. 3a, b). Specifically, *Nba*Ago.1 and *Nba*Ago.2 are connected via the PIWI–PIWI interface, *Nba*Ago.2 and *Nba*Ago.3 via the MID–PIWI and MID–MID interfaces, *Nba*Ago.3 and *Nba*Ago.4 via the PIWI–PIWI interface, and so on (Fig. 3b). At the PIWI–PIWI interface, K324.1 forms charged interaction with E360.2, R287.1 forms charged interaction with E253.2, and R285.2 forms cation–π interaction with F256.1 and polar contact with the main chain of G254.1 (Fig. 3d). At the MID–PIWI interface, Q134.3 forms polar contact with R295.2, while R142.3 forms charged interaction with D480.2 (Fig. 3e). At the MID–MID interface, the side chain of R244.3 forms polar contacts with the main chains of G140.2 and R142.2 and the side chain of E97.2 (Fig. 3e). Notably, the Ago–Ago dimerization interactions observed in the guide-bound complex are distinct from those involved in filament formation in the active complex (Figs. 2e, 3d). Group alanine substitution of interacting amino acids at the MID–MID and MID–PIWI interfaces impaired the collateral nuclease activity (Fig. 3h). Notably, group alanine substitution of interacting residues at the PIWI–PIWI interface disrupted filament formation, abolished collateral nuclease activity, and eliminated the defense against invading DNA as revealed by bacteriophage infection and plasmid interference assays (Fig. 3g–i; Supplementary information, Fig. S6c).

To investigate why only the longer tDNA induces filament formation, we compared the structures of guide-bound, active, and inactive complexes (Fig. 3f). From structures of active and inactive complexes, we observed that *Nba*Ago, together with the APAZ domain, creates a positively charged channel to accommodate the B-form guide–target heteroduplex, forming extensive interactions (Fig. 3c). Propagation of the guide–target heteroduplex leads to a dramatic conformational rearrangement around the PIWI domain, particularly the “sensor loop” (aa 280–307), which is pushed outward in the active complex to form the PIWI–PIWI interface that is required for filament formation (Fig. 3f). Notably, the counterpart of the sensor loop in long pAgos (termed Loop L2 in *Tt*Ago) plays essential roles in the activation of target cleavage.^33^ Given this structural observation, the compromised filament formation and collateral nuclease activity caused by mismatches at the central region of the guide–target heteroduplex (position 9–14, Fig. 1d) may be attributed to the perturbation in the interactions between the sensor loop and the guide–target heteroduplex.

### Activation of nuclease activity in the DREN domain

**Fig. 4:**
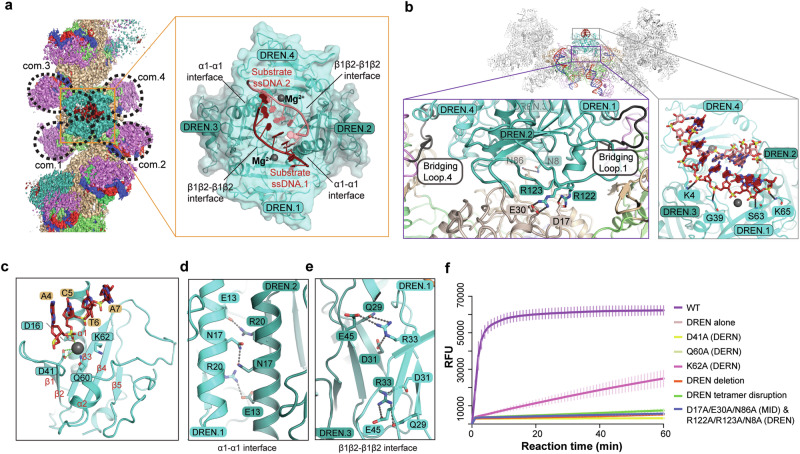
Activation mechanism of the DREN nuclease domain. **a** Cryo-EM map of the substrate-bound *Nba*SPARDA complex, with the DREN tetramer shown alongside in zoomed-in view. **b** Interaction details between the DREN tetramer and the filament (left), and interaction details between the main chains of DREN and the phosphate backbone of the substrate DNA (right). Bridging loops were colored in black. **c** Detailed structure of the DREN domain, and its interaction with the substrate ssDNA. **d** Interaction details on the α1–α1 interface. **e** Interaction details on the β1β2–β1β2 interface. **f** FQ assay to evaluate the effect of DREN-related mutants on collateral nuclease activity. FQ assay was performed in triplicate, and the error bars represent the standard deviations. DREN tetramer disruption mutant: N17A/E13A/R20A/R33A/Q29A/E45A/D31A.

The nuclease domain DREN, connected to the APAZ domain via a flexible loop without inter-domain contact, exhibits significant conformational diversity during the activation process. In the structures of the guide-bound and inactive complexes, DREN domains are flexible; however, it becomes stabilized by forming tetramers with D2 symmetry at the inner layers of the active and substrate-bound complex filaments (Figs. 3a, b, 4a). SEC experiment indicated that DREN deletion did not compromise the filament formation (Supplementary information, Fig. S7b), while the self-association of DREN depends on the filament formation of *Nba*Ago (Supplementary information, Fig. S1e). SEC-MALS (multi-angle light scattering with size exclusion chromatography) experiment indicated that the DREN domain alone is monomeric in solution, with an estimated molecular weight of 20 kDa (Supplementary information, Fig. S7a, c). FQ assay showed that the DREN domain alone exhibited undetectable nuclease activity (Fig. 4f). Collectively, these observations indicate that the tetramerization of the DREN domains is induced via the filament formation, and the collateral nuclease activity is activated via DREN tetramerization.

In the inner layer, the individual domains of the DREN tetramer are derived from four neighboring *Nba*SPARDA complexes, with DREN-APAZ.2 and DREN-APAZ.3 extending the bridging loop, while DREN-APAZ.1 and DREN-APAZ.4 folding it (Fig. 4a, b). The bridging loop is sandwiched between the DREN domain and the APAZ domain, anchoring the DREN tetramer to the filament (Fig. 4b). The bridging loop proved to be important, as deletion or extension of the bridging loop abolished the collateral nuclease activity (Fig. 3h). In addition, the inter-domain interactions (D17^MID^–R122^DREN^, E30^MID^–R123^DREN^, and N86^MID^–N8^DREN^) were observed to stabilize the DREN tetramer on the filament (Fig. 4b), and group alanine substitution of these interacting residues abolished the collateral nuclease activity (Fig. 4f). In the substrate-bound complex, the catalytic pockets of DREN.2 and DREN.3 face towards the filament, while the catalytic pockets of DREN.1 and DREN.4 face towards the solvent and are bound with two substrate ssDNA molecules (ssDNA.1 and ssDNA.2), respectively (Fig. 4a). Interestingly, DALI search revealed that DREN resembles the homing endonuclease I-Ssp6803I, which also forms a tetramer of D2 symmetry to catalyze the cleavage of pseudo-palindromic dsDNA (Supplementary information, Fig. S7d).^29^

Detailed structural analysis of the substrate-bound complex revealed that the DREN domain adopts a PD-(D/E)-XK fold (αβββαβ), with β-sheets flanked by the N-terminal helix α1 on one side and a short helix α2 (aa 83–91) on the other side (Fig. 4c). The catalytic residues, D41 (β2), Q60 (β3), and K62 (β3), align on the surface of the DREN domain to accommodate the substrate ssDNA for cleavage (Fig. 4c). D41 and Q60 coordinate a magnesium ion that points to the potential scissile phosphate group (Fig. 4c). Loading of substrate ssDNA causes minimal structural changes in DREN, with an RMSD (root-mean-square deviation) of 0.8 (Supplementary information, Fig. S7e). Alanine substitution of the catalytic residue D41 abolished the collateral nuclease activity in vitro and the defense against invading DNA in vivo (Figs. 3h, 4f). Q60A abolished the collateral nuclease activity, and K62A impaired the collateral nuclease activity (Fig. 4f). DREN tetramerization is mediated by interactions at α1–α1 and β1β2–β1β2 interfaces (Fig. 4a). At the α1–α1 interface, two N17 residues form polar interactions, and E13 forms charged interactions with R20 (Fig. 4d). At the β1β2–β1β2 interface, R33 forms polar and charged interactions with Q29, E45, and D31 (Fig. 4e). Group alanine substitution of these interacting residues abolished the collateral nuclease activity in vitro and the defense against invading DNA in vivo (Figs. 3i, 4f). DREN tetramerization likely triggers the collateral nuclease activity through the following mechanisms. Firstly, although cleavage of each ssDNA molecule is catalyzed by either DREN.1 or DREN.4, each ssDNA molecule is stabilized by both DREN.1 and DREN.4 through interactions between main chains and phosphate backbones. For instance, ssDNA.1 contacts the main chains of K65, S63, and G39 from DREN.1 and K4 from DREN.4 (Fig. 4b). Secondly, DREN tetramer creates a positively charged and symmetrical pocket to accommodate two ssDNA molecules to form a pseudo-duplex, allowing formation of promiscuous base interactions (such as base stacking, wobble pairs or base pairs) between them (Fig. 4b; Supplementary information, Fig. S7e, f). Collectively, these findings explained why tetramerization is required for DREN to activate collateral nuclease activity.

### Working model of *Nba*SPARDA complex

**Fig. 5:**
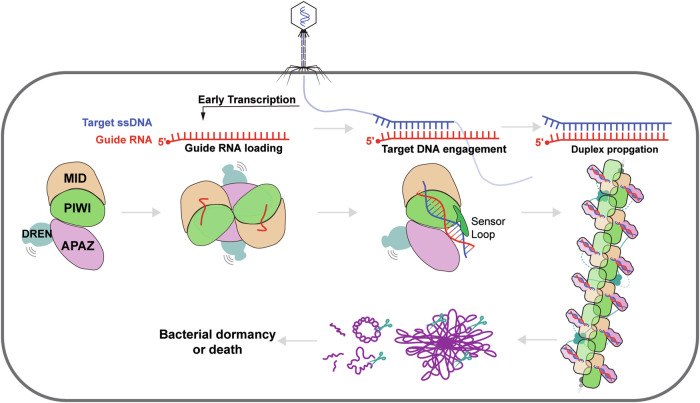
Working model of *Nba*SPARDA complex. *Nba*SPARDA complex senses the invasion of bacteriophage, goes through the “monomer-dimer-monomer-filament” transition, and activates its collateral nuclease activity against invading DNA and genomic DNA to cause bacterial death or dormancy.

Collectively, these experimental results led to a detailed working model of *Nba*SPARDA that highlights the importance of the transition in assembly form during the activation process (Fig. 5). gRNA loading, tDNA engagement, and guide–target heteroduplex propagation lead to the “monomer-dimer-monomer-filament” transition of SPARDA complex. During this process, the DREN domain is flexible but eventually stabilized by the formation of tetramers on the filament, which unleashes its collateral nuclease activity. The multi-turnover collateral nuclease activity of the DREN domain would lead to the enduring degradation of the host genome and invading DNA, ultimately resulting in cell death or dormancy.

## Discussion

The collateral nuclease activity of SPARDA makes it an effective host defense system and a promising tool for nucleic acid detection. The most interesting finding of this study lies in the target-induced filament formation of *Nba*SPARDA complex and filament-induced tetramerization of the DREN nuclease domain, providing an unexpected working mechanism distinct from those of SPARTA and SPARSA, and also different from the existing working model of SPARDA.^28^ For the first time, Ago was found to form filaments to exert its function. However, whether the filament formation mechanism is conserved in other SPARDA systems remains to be studied.

Transition of assembly form plays a crucial role in various cellular events. Oligomerization in various forms, especially high-order assembly, proved to be important in mammalian and prokaryotic host defense. In the metazoan cGAS-STING immunity pathway, STING binds cyclic di-AMP and forms filaments, activating subsequent signaling pathways.^34^ In the bacterial defense system, Thoeris, antiphage immunity is conferred through filament assembly of the SIR2 effector.^35^ Similar mechanisms have been reported in recent studies in pAgos. Our previous studies on thermophilic long pAgos indicated that tDNA engagement induces dimerization and activation, and that dimerization stabilizes the catalytic loops to facilitate target cleavage.^36^ Similarly, tDNA induces the tetramerization of the SPARTA and SPARSA complexes to activate their NADase activity.^26^ This study on SPARDA reinforces the emerging theme regarding how the transition of assembly form, especially the high-order assembly, dictates the enzymatic activity of Agos.

Despite the diversity in the Ago family, remote similarities are evident when comparing *Nba*SPARDA with other short Ago systems. In SPARDA, the APAZ domain plays a similar role to the packing-type N domain, as observed in *Rhodobacter sphaeroides* Ago, facilitating the guide–target heteroduplex propagation without blocking further base-pairing between the guide and target.^37^ Nucleases from the PD-(D/E)-XK superfamily are not only utilized by the SPARDA system. A recent study also highlighted their cooperation with long-B pAgos which lack catalytic tetrad and nuclease activity.^38^ Sensor loops were found to be important in triggering the transition of assembly form in SPARTA, SPARSA, and SPARDA. Intriguingly, the counterpart of the sensor loop in long pAgos (termed Loop L1/L2/L3) undergoes similar arrangement to activate the target cleavage activity during the guide–target duplex propagation.^33^ This implied that, despite the loss of catalytic residues, PIWI domains preserve their regulatory roles across both long and short pAgos.

A critical and interesting question is why tetramerization of the DREN nuclease domain is required to exhibit collateral nuclease activity. Although DREN tetramers are structurally similar to the homing endonuclease I-Ssp6803I tetramers, DREN should presumably have no substrate sequence preference, given its collateral nuclease activity. Although the sequence of substrate ssDNA is not palindromic, two ssDNA molecules still form a pseudo-duplex, and this symmetry-forced pseudo-duplex formation likely stabilizes both substrate ssDNA molecules to promote the collateral cleavage activity. Due to the resolution limitations, we cannot exclude the possibility that tetramerization induces subtle conformational changes in the catalytic site to trigger the collateral nuclease activity. Besides, due to lack of apo structure, it remains not understood why gRNA loading leads to dimerization of *Nba*SPARDA complex.

## Materials and methods

### Molecular cloning and mutagenesis

His-tagged *Nba*Ago and strep-tagged DREN-APAZ were cloned into the first and second multiple cloning sites of pRSFDuet-1, respectively. Both protein-coding sequences were codon-optimized and sequencing-verified for expression in *Escherichia coli*. The point mutations were generated using the QuickChange method. Large-fragment deletion was generated by ligating the backbone, amplified through inverse PCR, with DNA oligonucleotides.

### Protein expression and purification

*E. coli* BL21(DE3) cells freshly transformed with the *Nba*SPARDA were cultured in TB medium containing 30 μg/mL kanamycin at 37 °C with shaking (220 rpm) until the OD_600_ reached 0.6–0.8. Protein expression was induced at 18 °C by 0.3 mM IPTG. The cells were collected after 18 h and resuspended in buffer A containing 20 mM Tris-HCl (pH 8.0), 500 mM NaCl, 20 mM imidazole, 2 mM DTT, 1 mM PMSF, and 2% glycerol. After being lysed by sonication and centrifugation (14,000 rpm, 30 min) at 4 °C, the supernatant was loaded onto Ni-NTA resin pre-equilibrated with lysis buffer A. After being washed with additional buffer A, the His-tagged proteins were eluted with buffer B containing 20 mM Tris-HCl (pH 8.0), 500 mM NaCl, 500 mM imidazole, 2 mM DTT, and 2% glycerol. Subsequently, the eluate was diluted 3-fold with buffer C containing 20 mM Tris-HCl (pH 8.0), 2 mM DTT, and 2% glycerol. The diluted sample was supplemented with 5 mM EDTA and further purified using a HiTrap Heparin HP column (Cytiva) pre-equilibrated with buffer D containing 20 mM Tris-HCl (pH 8.0), 150 mM NaCl, and 2 mM DTT. After being washed with additional buffer D, the protein was eluted using a NaCl gradient (from 150 mM to 800 mM) in buffer E containing 20 mM Tris-HCl (pH 8.0), 1 M NaCl, and 2 mM DTT. The peak fractions containing the protein complex were pooled, analyzed by SDS-PAGE, concentrated and fractionated on a Superdex 200 column (Cytiva) with buffer F containing 20 mM Tris-HCl (pH 8.0), 200 mM NaCl, 2 mM DTT, and 2 mM MgCl_2_.

For reconstitution of the SPARDA–gRNA complex, the SPARDA was incubated with 20-nt 5′-phosphorylated gRNA at a molar ratio of 1:1.2 at 25 °C for 15 min. For reconstitution of the SPARDA–gRNA–tDNA complex, the 21-nt tDNA was then added at a molar ratio of 1:1.2:1.2 and incubated at 25 °C for another 15 min. The resulting sample was subsequently subjected to a Superdex 200 column with buffer F. The peak fractions were used to perform cryo-EM experiments.

For SEC-MALS, the active complex sample (*Nba*SPARDA–gRNA–tDNA (21-nt), 3 mg/mL in 200 µL) or the DREN-only sample (3.5 mg/mL in 200 µL) was loaded onto a buffer F pre-equilibrated Superdex 200 Increase 10/300 column (Cytiva) operated on a ProStar335 system (Varian) and a miniDAWN detector (Wyatt Technology) at a flow rate of 0.4 mL/min. Elution profiles were recorded by absorbance at 280 nm. The data were analyzed using ASTRA 6.0 (Wyatt Technology).

### FQ-labeled reporter assays

The fluorescence assay of *Nba*SPARDA activity was based on a previous protocol with minor modifications.^28^ *Nba*SPARDA (wild-type or mutants) at 800 nM was mixed with 600 nM gRNA for 15 min at 25 °C in buffer G containing 20 mM Tris-HCl (pH 8.0), 50 mM NaCl, 5 µg/mL BSA, 2 mM DTT, and 5 mM MgCl_2_. After incubation with 600 nM tDNA at 25 °C for 15 min, the sample was cooled on ice. Subsequently, 600 nM ssDNA substrate (BHQ2-GTCACAGAGATACTACGTGTGCGACTGCTCAG-ROX) was added. 20-µL aliquots of the samples were then transferred into 0.2 mL white 8-tube PCR strips. The samples were tested in triplicate, and the fluorescence was monitored using a 96-well real-time PCR detection system (CFX96 qPCR machine, Bio-Rad), with measurements taken every 1 min at 30 °C for 60 min.

### Plaque assays

The pRSFDuet-1 *Nba*SPARDA (wild-type or mutants) or empty pRSFDuet-1 was transformed into *E. coli* BL21(DE3). A single colony from an LB agar plate was grown in LB broth with ampicillin (100 µg/mL) at 37 °C to an OD_600_ of ~0.2. Protein expression was induced with 0.3 mM IPTG and further grown to an OD_600_ of 0.7–0.8. Subsequently, 500 µL of the culture was mixed with 15 mL of 0.5% LB top agar and poured onto LB plates with 100 µg/mL ampicillin and 0.2 mM IPTG. The plates were spotted with 1 µL of T5 bacteriophage diluted across five 10-fold dilutions (10^–1^ to 10^–5^). After overnight incubation at 30 °C, the plates were imaged.

### Plasmid interference assays

*E. coli* BL21(DE3) was transformed with pCDFDuet-1 and pRSFDuet-1 (empty, *Nba*SPARDA wild-type or mutants). A single colony from an LB agar plate was cultured in LB broth with Kanamycin (50 µg/mL) and Spectinomycin (50 µg/mL) at 37 °C to an OD_600_ of ~0.2. Protein expression was induced with 0.3 mM IPTG, and the culture was grown to an OD_600_ of 0.7–0.8. The plates containing Kanamycin, Spectinomycin, and IPTG were spotted with 1 µL of culture diluted across six 10-fold dilutions (10^–1^ to 10^–6^). The plates were imaged after overnight incubation at 30 °C.

### Bimolecular fluorescence complementation assays

*Nba*Ago and sfGFP (N)-DREN-APAZ were cloned into the first and second multiple cloning sites of pRSFDuet-1. *Nba*Ago and sfGFP (C)-DREN-APAZ were cloned into the corresponding sites of pETDuet-1. pCDFDuet-1 (interference plasmid), pETDuet-1, and pRSFDuet-1were co-transformed into *E. coli* BL21(DE3). Colonies selected from LB agar plates were grown in LB broth supplemented with Ampicillin (100 μg/mL), Kanamycin (50 μg/mL), and Spectinomycin (50 μg/mL) at 37 °C. Cells were cultured until an OD_600_ of ~0.6 was reached, induced with 0.3 mM IPTG at 18 °C, harvested after 16 h, and then resuspended in PBS buffer. Samples were imaged using a ZEISS LSM 900 confocal laser scanning microscope (CLSM, Carl Zeiss) equipped with a 63× oil immersion objective. Images were taken at an excitation wavelength of 507 nm, and contrast and brightness settings for each channel were individually adjusted using the ZEN Microscopy software (Carl Zeiss).

### Electron microscopy

The concentrations of guide-bound, inactive, active, and substrate-bound *Nba*SPARDA complex were 0.4 mg/mL, 0.45 mg/mL, 0.2 mg/mL and 0.16 mg/mL, respectively. For all protein samples, 4-μL aliquots were applied to glow-discharged UltrAuFoil holey gold grids (R1.2/1.3, 300 mesh). The grids were plunged into liquid ethane using a Vitrobot. The substrate-bound complex was blotted with force 3 for 12 s, and the others were blotted with force 3 for 7 s. Cryo-EM data were collected with a Titan Krios microscope (FEI) operated at 300 kV and images were collected using EPU^39^ at a nominal magnification of 105,000× (resulting in a calibrated physical pixel size of 0.85 Å/pixel) with a defocus range from 1.2 μm to 2.2 μm. The images were recorded on a K3 summit electron direct detector in super-resolution mode at the end of a GIF-Quantum energy filter operated with a slit width of 20 eV. A dose rate of 15 electrons per pixel per second and an exposure time of 2.5 s were used, generating 40 movie frames with a total dose of ~54 electrons per Å^2^. A total of 1971, 2930, 2249, and 2053 movie stacks were collected for guide-bound, inactive, active, and substrate-bound *Nba*SPARDA complex, respectively (Supplemental information, Table S1).

### Cryo-EM image processing

The movie frames were imported to RELION-3, aligned using MotionCor2^40^ with a binning factor of 2, and subjected to contrast transfer function estimation using Gctf^41^ on the fly.

For guide-bound *Nba*SPARDA complex, 1971 movies were collected. 2,171,204 particles were auto-picked with templates. 619,615 particles were selected after 2D classification. 295,471 particles were selected after heterogeneous refinement,^42^ and used for final auto-refinement with C2 symmetry, converging at a resolution of 2.93 Å. For inactive *Nba*SPARDA complex, 2930 movies were collected. 4,918,325 particles were auto-picked with templates. 1,703,577 particles were selected after 2D classification. 213,852 particles were selected after two rounds of 3D classification, and used for final non-uniform refinement without symmetry, converging at a resolution of 3.34 Å. For active *Nba*SPARDA complex, 2249 movies were collected. 605,499 particles were auto-picked with templates. 55,611 particles were selected for heterogeneous refinement after 2D classification. 39,659 particles were selected after heterogeneous refinement, and used for final non-uniform refinement without symmetry, converging at a resolution of 3.18 Å. The filament shows helical symmetry with a twist of 87° and a rise of 121.72 Å. The resolution (3.4 Å) of reconstruction using helical symmetry was lower than the resolution of reconstruction without symmetry (3.18 Å), probably due to the curvature and heterogeneity in length. For substrate-bound *Nba*SPARDA complex, 2053 movies were collected. 583,402 particles were auto-picked with templates. 477,105 particles were selected after 2D classification. 148,359 particles were selected after 3D classification, and used for final auto-refinement without symmetry, converging at a resolution of 3.19 Å.

### Structure analysis, model building, and visualization

For model building of the guide-bound and active *Nba*SPARDA complex, structures of individual protein subunits were predicted via AlphaFold2^43^ and manually adjusted in Coot,^44^ and the structures of nucleic acids were built de novo in Coot. For model building of the substrate-bound complex, the substrate ssDNA was built de novo in Coot, while others were modeled based on the structure of the active complex with manual adjustment in Coot. For model building of the inactive complex, protein subunits were built based on the structure of the guide-bound complex with manual adjustment in Coot, and the nucleic acids were built de novo in Coot. Model refinement was performed by the *phenix.real_space_refine* tool in Phenix.^45^ Figures and movies were generated by PyMOL and UCSF Chimera.^46^

## Supplementary information

Supplementary information, Fig. S1

Supplementary information, Fig. S2

Supplementary information, Fig. S3

Supplementary information, Fig. S4

Supplementary information, Fig. S5

Supplementary information, Fig. S6

Supplementary information, Fig. S7

Supplementary information, Table S1

## Data Availability

Cryo-EM reconstructions of the guide-bound, inactive, active, and substrate-bound *Nba*SPARDA complexes have been deposited in the Electron Microscopy Data Bank under the accession numbers EMD-61769, EMD-61780, EMD-61787 and EMD-61790, respectively. Coordinates for atomic models of the guide-bound, inactive, active and substrate-bound *Nba*SPARDA complexes have been deposited in the Protein Data Bank under the accession numbers 9JSB, 9JSP, 9JSZ and 9JT2, respectively.
