# Supplementary material for: Target DNA-induced filament formation and nuclease activation of SPARDA complex
Source: Cell Res. 2025 Mar 24;35(7):510–9. doi: 10.1038/s41422-025-01100-z (PMC12205087; doi:10.1038/s41422-025-01100-z)
Supplement: Supplementary file 1 — Supplementary information, Fig. S1 [file 41422_2025_1100_MOESM1_ESM.pdf]

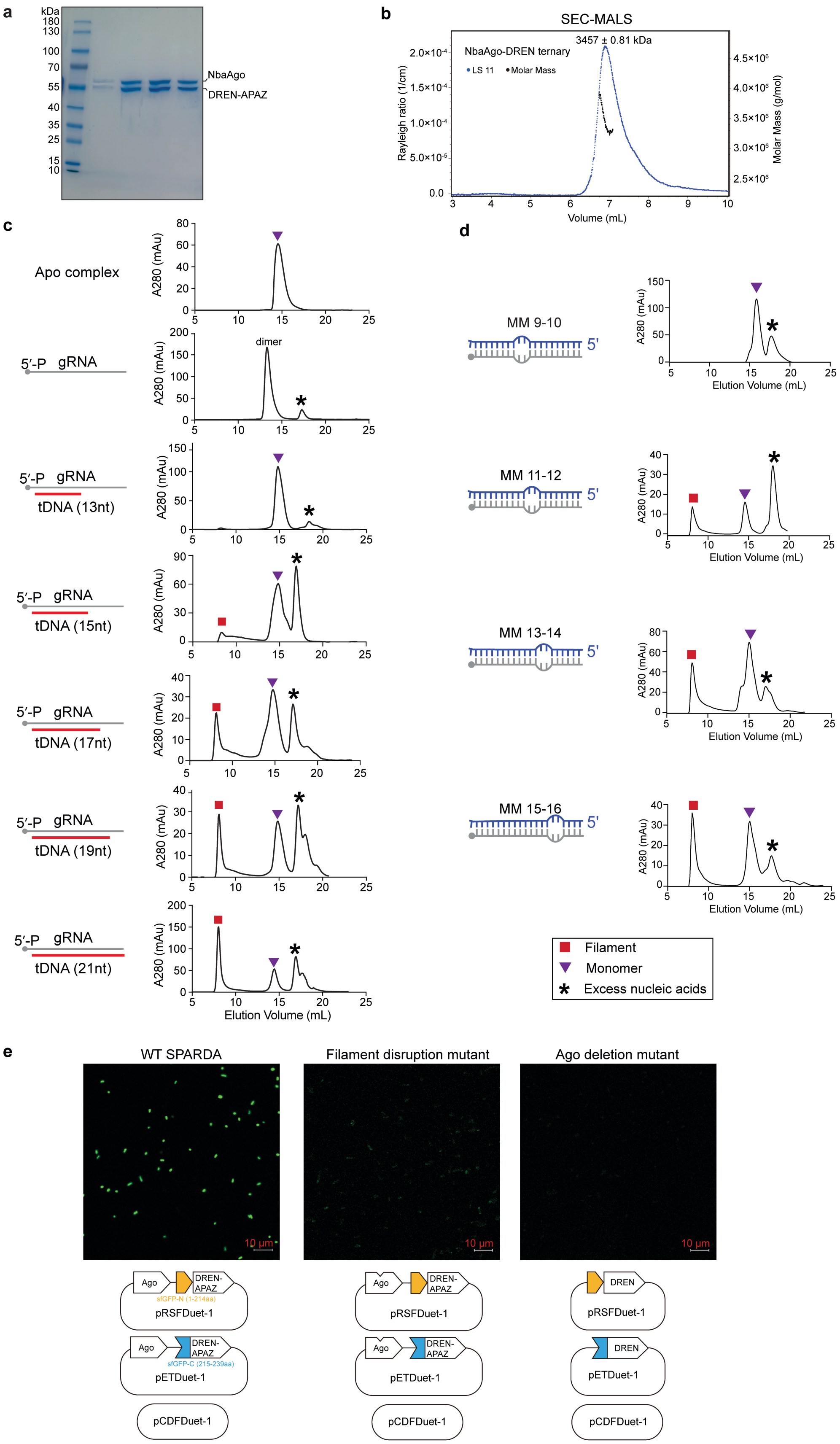

a
kDa
180
130
100
70
55
40
35
25
15
10
NbaAgo
DREN-APAZ
b
SEC-MALS
NbaAgo-DREN ternary
3457 ± 0.81 kDa
LS 11
Molar Mass
Rayleigh ratio (1/cm)
Molar Mass (g/mol)
Volume (mL)
c
Apo complex
5′-P gRNA
tDNA (13nt)
tDNA (15nt)
tDNA (17nt)
tDNA (19nt)
tDNA (21nt)
dimer
A280 (mAu)
Elution Volume (mL)
d
MM 9-10
MM 11-12
MM 13-14
MM 15-16
5'
A280 (mAu)
Elution Volume (mL)
Filament
Monomer
Excess nucleic acids
e
WT SPARDA
Filament disruption mutant
Ago deletion mutant
10 μm
Ago
DREN-APAZ
sfGFP-N (1-214aa)
sfGFP-C (215-239aa)
DREN
pRSFDuet-1
pETDuet-1
pCDFDuet-1

Figure S1. Biochemical characterization of *Nba*SPARDA complex. (a) SDS-PAGE of apo *Nba*SPARDA complex. **(b)** SEC-MALS profile of *Nba*SPARDA filament. **(c)** SEC profiles of *Nba*SPARDA complex, alone, loaded with guide RNA, and loaded with guide RNA and target DNA of varying length (13-, 15-, 17-, 19-, and 21-nt). **(d)** SEC profiles of *Nba*SPARDA complex loaded with guide RNA and target DNA containing mismatches at different positions (9-10, 11-12, 13-14, and 15-16). **(e)** BiFC experiment to validate the filament formation of SPARDA complex. The N-split (a.a. 1-214) and C-split (a.a. 215-239 aa) sfGFP were respectively fused to the N-terminus of DREN, and the pCDFDuet-1 was used as an interference plasmid to activate SPADRA. Filament disruption mutations include R287A/E253A/ K324A/E360A/R285A/F256A of Ago.
