# Supplementary material for: Target DNA-induced filament formation and nuclease activation of SPARDA complex
Source: Cell Res. 2025 Mar 24;35(7):510–9. doi: 10.1038/s41422-025-01100-z (PMC12205087; doi:10.1038/s41422-025-01100-z)
Supplement: Supplementary file 2 — Supplementary information, Fig. S2 [file 41422_2025_1100_MOESM2_ESM.pdf]

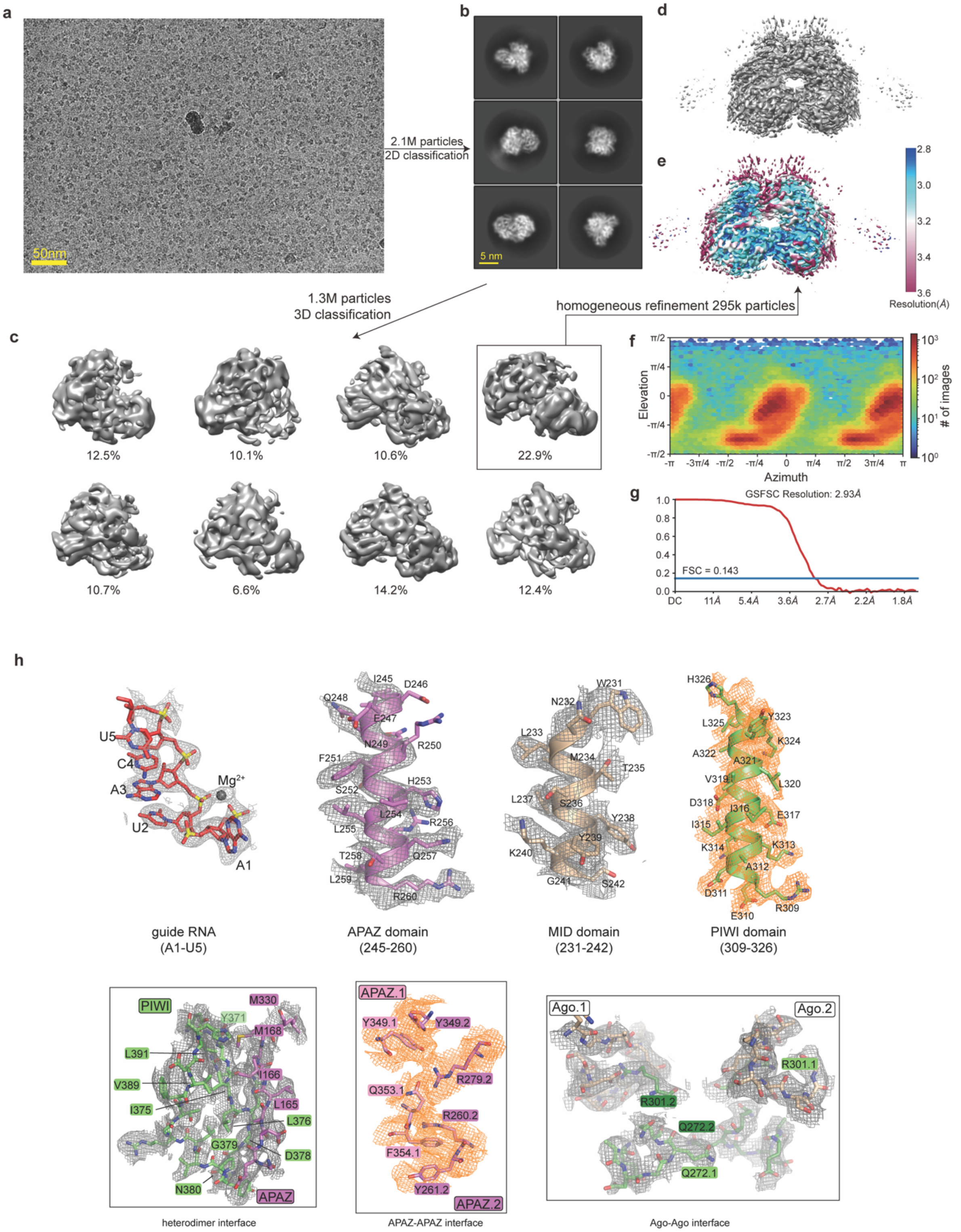

a
50nm
b
5 nm
2.1M particles
2D classification
d
e
2.8
3.0
3.2
3.4
3.6
Resolution(Å)
1.3M particles
3D classification
homogeneous refinement 295k particles
c
12.5%
10.1%
10.6%
22.9%
10.7%
6.6%
14.2%
12.4%
f
Elevation
Azimuth
# of images
g
GSFSC Resolution: 2.93Å
FSC = 0.143
DC
11Å
5.4Å
3.6Å
2.7Å
2.2Å
1.8Å
h
U5
C4
A3
U2
A1
Mg2+
guide RNA
(A1-U5)
APAZ domain
(245-260)
MID domain
(231-242)
PIWI domain
(309-326)
PIWI
APAZ
heterodimer interface
APAZ.1
APAZ.2
APAZ-APAZ interface
Ago.1
Ago.2
Ago-Ago interface

Figure S2. Cryo-EM of guide-bound *Nba*SPARDA complex. (a) A representative raw cryo-EM micrograph of the gRNA-bound complex. **(b)** Representative 2D class averages. **(c)** 3D classification. **(d)** Cryo-EM map of consensus refinement. **(e)** Local resolution estimation of the reconstruction in **d**. **(f)** Angular distribution of the reconstruction in **d**. **(g)** FSC (Fourier shell correlation) plots of the reconstruction in **d**. **(h)** Cryo-EM density of guide RNA, representative helices, and dimerization residues from guide-bound complex with atomic models fitted in.
