# Supplementary material for: Target DNA-induced filament formation and nuclease activation of SPARDA complex
Source: Cell Res. 2025 Mar 24;35(7):510–9. doi: 10.1038/s41422-025-01100-z (PMC12205087; doi:10.1038/s41422-025-01100-z)
Supplement: Supplementary file 3 — Supplementary information, Fig. S3 [file 41422_2025_1100_MOESM3_ESM.pdf]

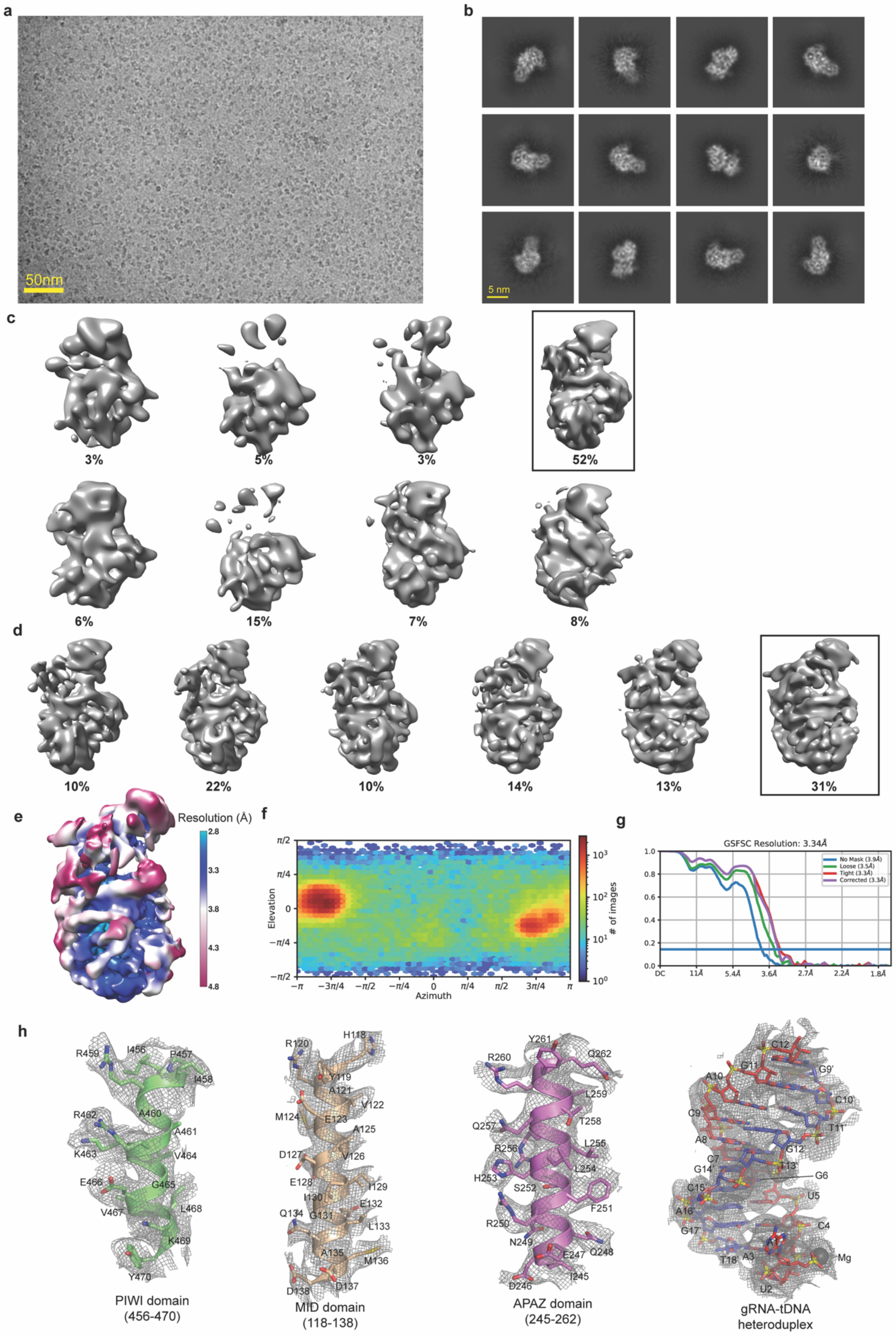
a
50nm
b
5 nm
c
3%
5%
3%
52%
6%
15%
7%
8%
d
10%
22%
10%
14%
13%
31%
e
Resolution (Å)
2.8
3.3
3.8
4.3
4.8
f
Elevation
Azimuth
# of images
g
GSFSC Resolution: 3.34Å
No Mask (3.9Å)
Loose (3.5Å)
Tight (3.3Å)
Corrected (3.3Å)
h
PIWI domain
(456-470)
MID domain
(118-138)
APAZ domain
(245-262)
gRNA-tDNA
heteroduplex

Figure S3. Cryo-EM of the inactive *Nba*SPARDA complex. (a) A representative raw cryo-EM micrograph of the inactive complex. **(b)** Representative 2D class averages. **(c)** First round of 3D classification. **(d)** Second round of 3D classification. **(e)** Cryo-EM map of consensus refinement color based on local resolution estimation. **(f)** Angular distribution of the reconstruction in **e**. **(g)** FSC plots of the reconstruction in **e**. **(h)** Cryo-EM density of representative helices and heteroduplex of inactive complex with atomic models fitted in.
