# Supplementary material for: Target DNA-induced filament formation and nuclease activation of SPARDA complex
Source: Cell Res. 2025 Mar 24;35(7):510–9. doi: 10.1038/s41422-025-01100-z (PMC12205087; doi:10.1038/s41422-025-01100-z)
Supplement: Supplementary file 4 — Supplementary information, Fig. S4 [file 41422_2025_1100_MOESM4_ESM.pdf]

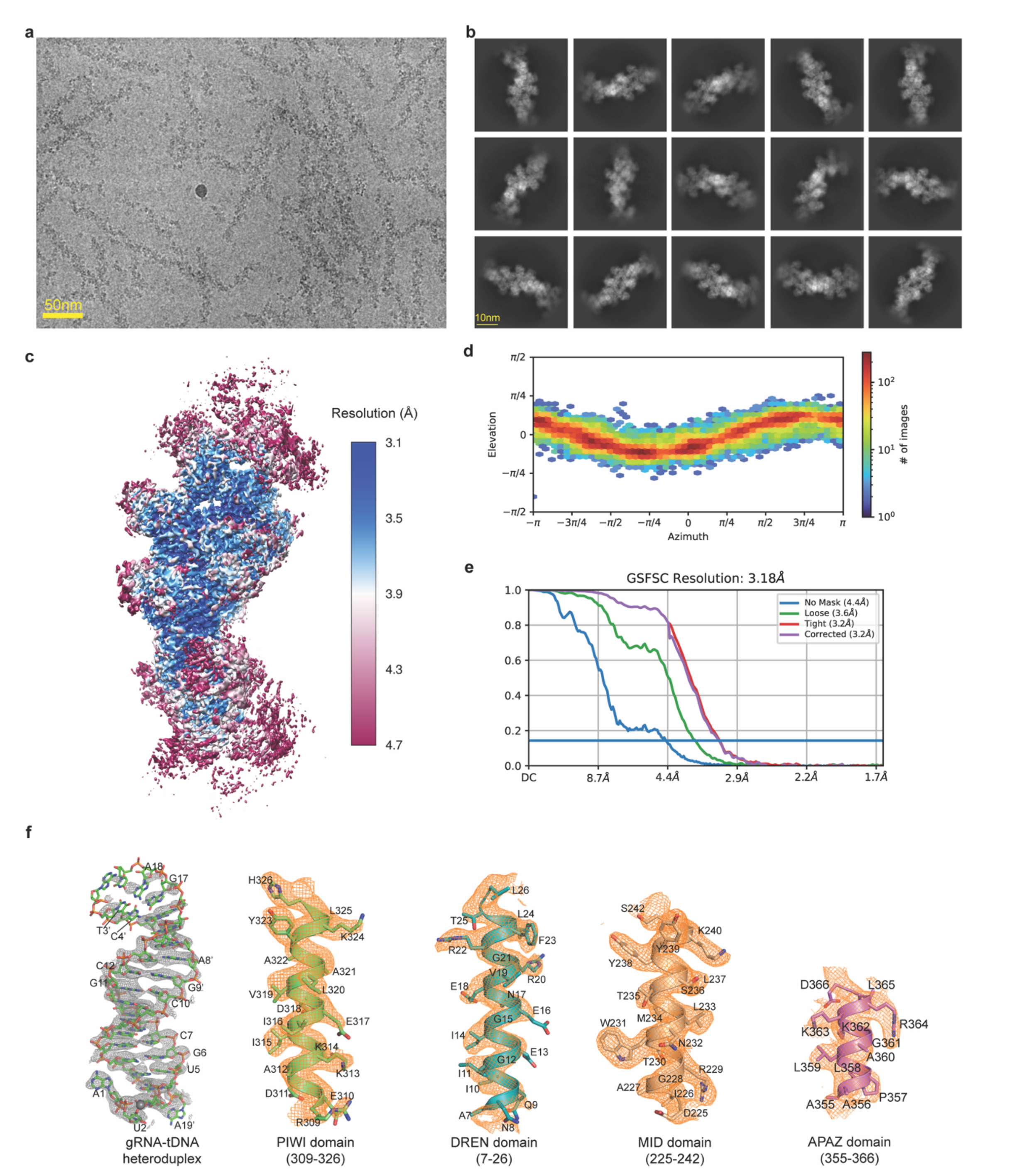

Figure S4. Cryo-EM of the active *Nba*SPARDA complex. **(a)** A representative raw cryo-EM micrograph of the active complex. **(b)** Representative 2D class averages. **(c)** Cryo-EM map of consensus refinement colored based on local resolution estimation. **(d)** Angular distribution of the reconstruction in **c**. **(e)** FSC plot of the reconstruction in **c**. **(f)** Cryo-EM density of representative helices of active complex with atomic models fitted in.
