# Supplementary material for: Target DNA-induced filament formation and nuclease activation of SPARDA complex
Source: Cell Res. 2025 Mar 24;35(7):510–9. doi: 10.1038/s41422-025-01100-z (PMC12205087; doi:10.1038/s41422-025-01100-z)
Supplement: Supplementary file 5 — Supplementary information, Fig. S5 [file 41422_2025_1100_MOESM5_ESM.pdf]

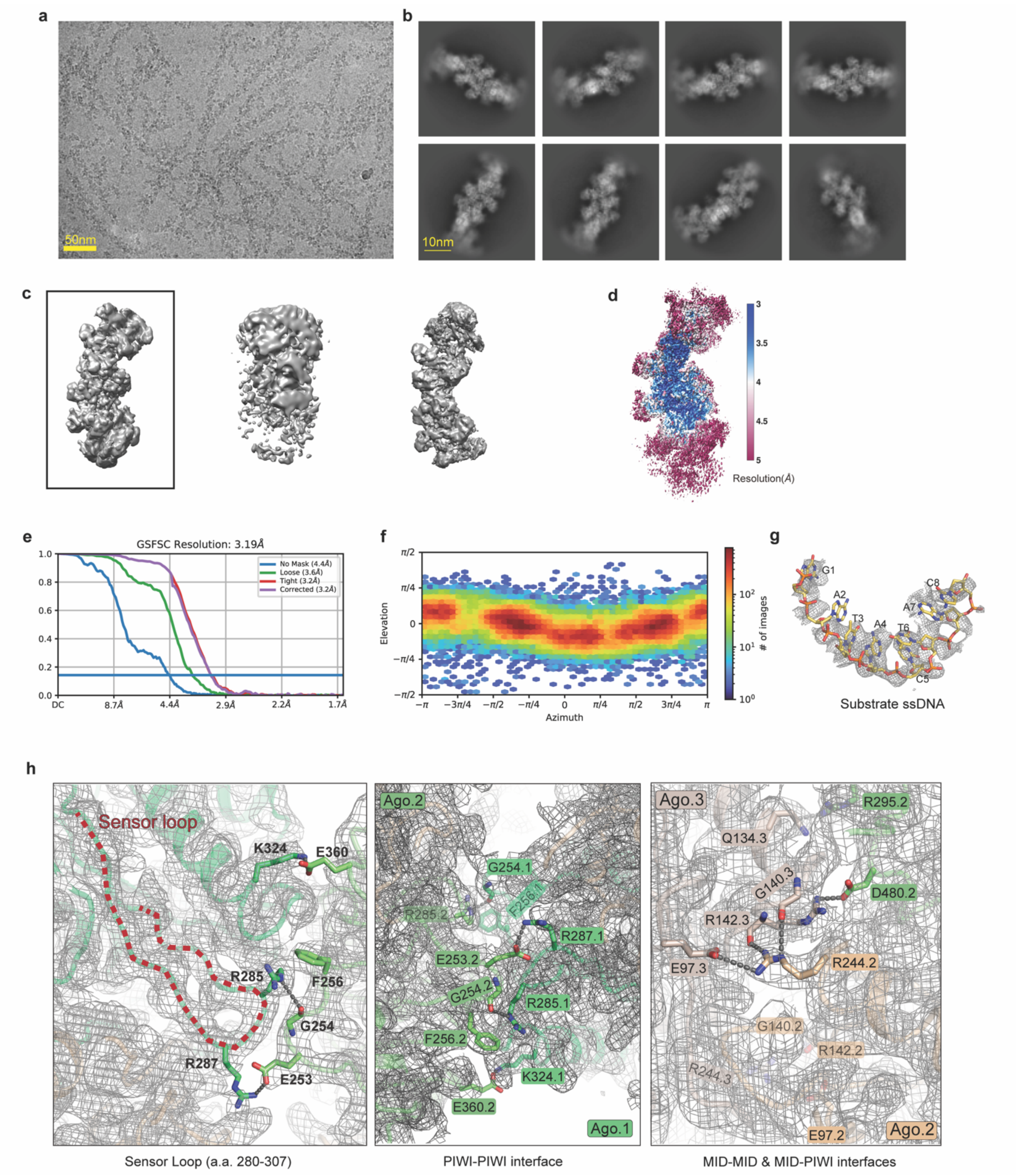

Figure S5. Cryo-EM of substrate-bound *Nba*SPARDA complex. **(a)** A representative raw cryo-EM micrograph of the substrate-bound complex. **(b)** Representative 2D class averages. **(c)** 3D classification. **(d)** Cryo-EM map of consensus refinement colored based on local resolution estimation. **(e)** Angular distribution of the reconstruction in **d**. **(f)** FSC plot of the reconstruction in **d**. **(g)** The cryo-EM density of substrate ssDNA from the substrate-bound complex. **(h)** The cryo-EM density of sensor loop and residues mediating the filament formation.
