# Supplementary material for: Target DNA-induced filament formation and nuclease activation of SPARDA complex
Source: Cell Res. 2025 Mar 24;35(7):510–9. doi: 10.1038/s41422-025-01100-z (PMC12205087; doi:10.1038/s41422-025-01100-z)
Supplement: Supplementary file 6 — Supplementary information, Fig. S6 [file 41422_2025_1100_MOESM6_ESM.pdf]

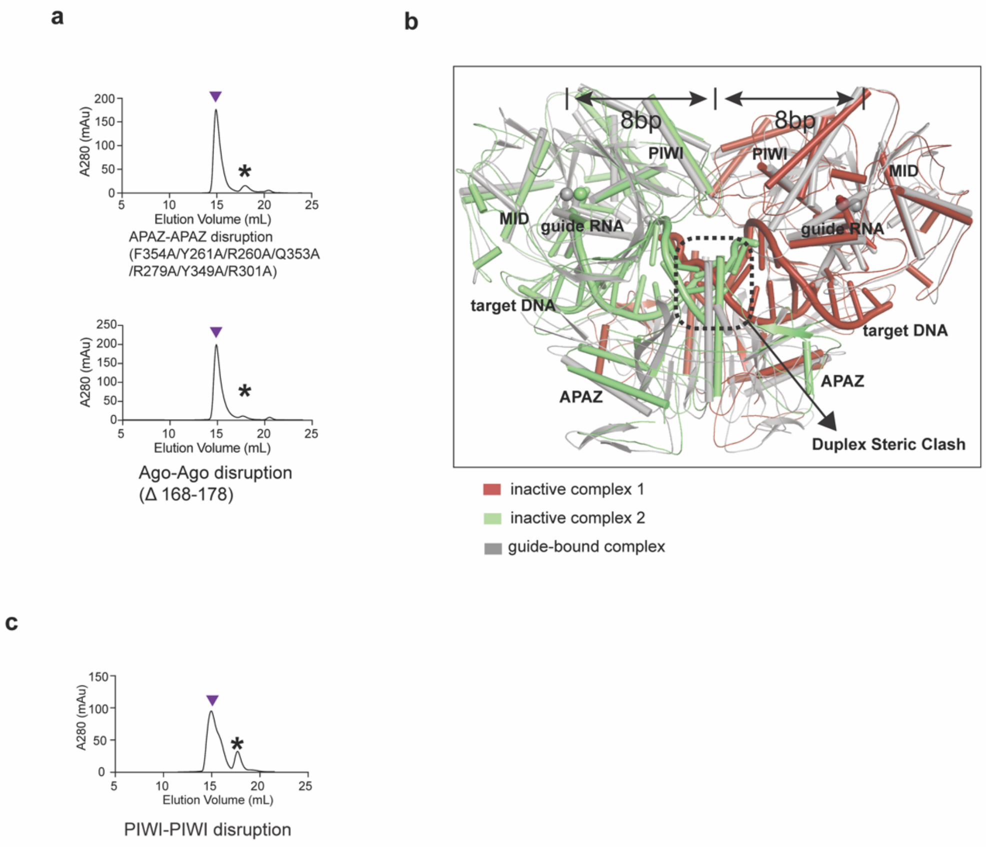

Figure S6. SEC of *Nba*SPARDA mutants and structural comparison between guide-bound and inactive complexes. (a) SEC profiles of *Nba*SPARDA complex mutants with dimerization interfaces disruption. **(b)** Superposition of two copies of inactive complexes into the structure of guide-bound complex. The superposition was based on the whole SPARDA protein complex. **(c)** SEC profile of *Nba*SPARDA complex with PIWI-PIWI disruption.
