# Supplementary material for: Target DNA-induced filament formation and nuclease activation of SPARDA complex
Source: Cell Res. 2025 Mar 24;35(7):510–9. doi: 10.1038/s41422-025-01100-z (PMC12205087; doi:10.1038/s41422-025-01100-z)
Supplement: Supplementary file 7 — Supplementary information, Fig. S7 [file 41422_2025_1100_MOESM7_ESM.pdf]

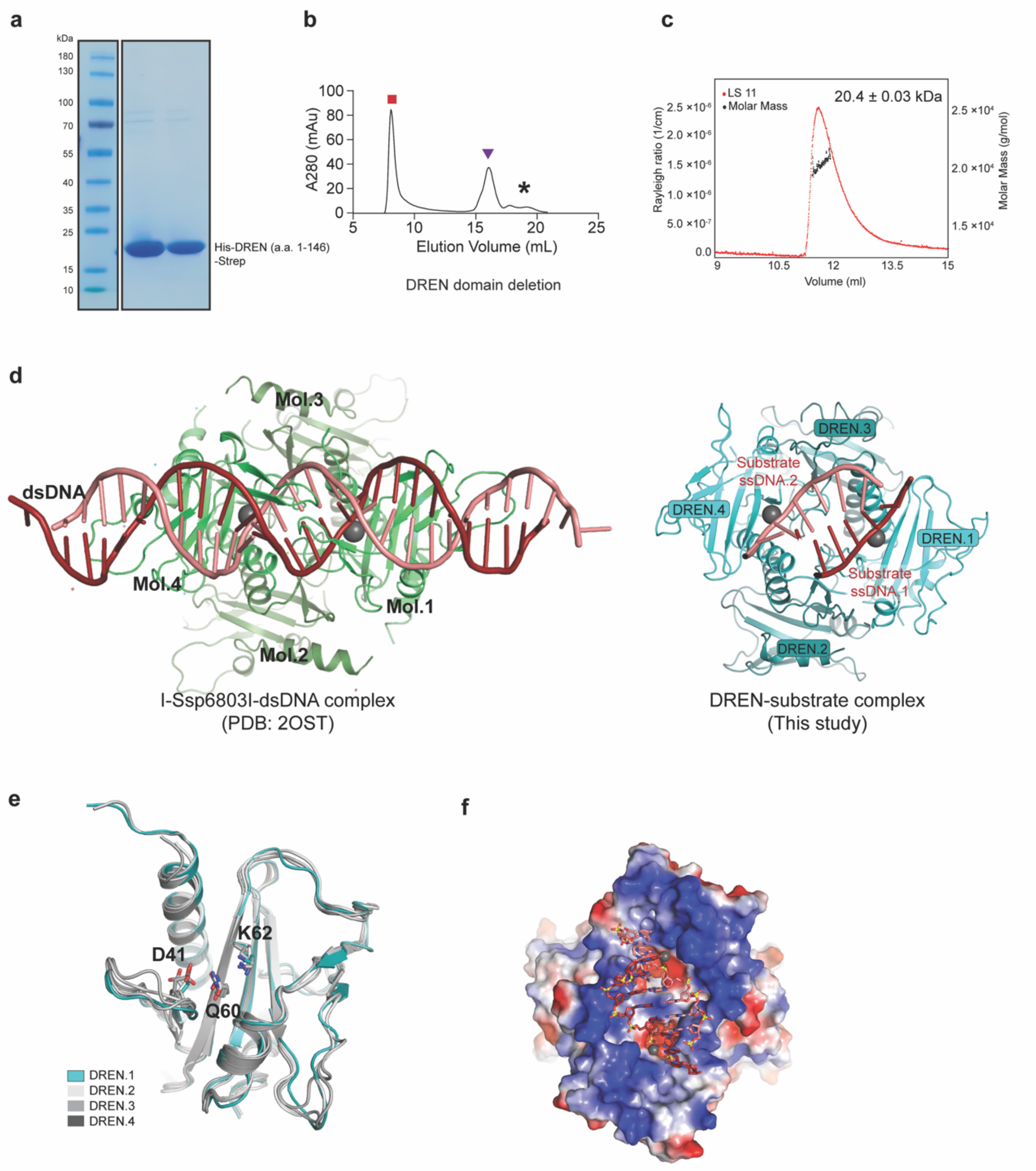

Figure S7. Biochemical and structural analysis of DREN domains. (a) SDS-PAGE of DREN domain. **(b)** SEC profile of *Nba*SPARDA complex with DREN domain deleted. **(c)** SEC-MALS profile to determine the molecular weight of DREN domain. **(d)** Structural similarity between I-Ssp6803I-dsDNA complex and DREN-substrate complex. I-Ssp6803I forms a tetramer to bind one dsDNA molecule. **(e)** Superposition of individual domains from a DREN tetramer bound with substrate ssDNA. **(f)** Electrostatic potential surface view of DREN-substrate ssDNA complex.
