# Supplementary material for: Target DNA-induced filament formation and nuclease activation of SPARDA complex
Source: Cell Res. 2025 Mar 24;35(7):510–9. doi: 10.1038/s41422-025-01100-z (PMC12205087; doi:10.1038/s41422-025-01100-z)
Supplement: Supplementary file 8 — Supplementary information, Table S1 [file 41422_2025_1100_MOESM8_ESM.pdf]

| | Guide-bound complex (9JSB, EMD-61769) | Inactive complex (9JSP, EMD-61780) | Active complex (9JSZ, EMD-61787) | Substrate-bound complex (9JT2, EMD-61790) |
|---|---|---|---|---|
| **Data collection and processing** | | | | |
| Magnification | 105,000 | 105,000 | 105,000 | 105,000 |
| Voltage (kV) | 300 | 300 | 300 | 300 |
| Electron exposure (e$^{-}$/Å$^{2}$) | 54 | 54 | 54 | 54 |
| Defocus range (μm) | 1.2 – 2.2 | 1.2 – 2.2 | 1.2 – 2.2 | 1.2 – 2.2 |
| Pixel size (Å) | 0.85 | 0.85 | 0.85 | 0.85 |
| Symmetry imposed | *C2* | *C1* | *C1* | *C1* |
| Initial particle images (no.) | 2,171,204 | 4,918,325 | 605,499 | 583,402 |
| Final particle images (no.) | 295,471 | 213,852 | 39,659 | 148,359 |
| Map resolution (Å) | 2.93 | 3.34 | 3.18 | 3.19 |
| FSC threshold | 0.143 | 0.143 | 0.143 | 0.143 |
| Map resolution range (Å) | 2.9-4.2 | 3.2-4.5 | 3.0-5.0 | 3.0-5.0 |
| | | | | |
| **Refinement** | | | | |
| Initial model used | AlphaFold2 | 9JSB | AlphaFold2 | 9JSZ |
| Model resolution (Å) | 3.2 | 3.5 | 3.5 | 3.4 |
| FSC threshold | 0.5 | 0.5 | 0.5 | 0.5 |
| Model resolution range (Å) | 2.8-3.5 | 3.2-3.9 | 3.2-3.8 | 3.1-3.5 |
| Map sharpening *B* factor (Å$^{2}$) | -150.7 | -173.3 | -69.3 | -92.7 |
| Model composition | | | | |
| Non-hydrogen atoms | 11436 | 5862 | 30776 | 31181 |
| Protein residues | 1404 | 673 | 3461 | 3463 |
| Nucleotides | 10 | 22 | 147 | 165 |
| *B* factors (Å$^{2}$) | | | | |
| Protein | 13.42 | 24.87 | 83.93 | 73.76 |
| Nucleotide | 5.58 | 23.41 | 97.57 | 110.05 |
| R.m.s. deviations | | | | |
| Bond lengths (Å) | 0.004 | 0.004 | 0.004 | 0.003 |
| Bond angles (°) | 0.781 | 0.825 | 0.768 | 0.693 |
| | | | | |
| **Validation** | | | | |
| MolProbity score | 2.15 | 1.97 | 2.00 | 1.91 |
| Clashscore | 7.84 | 6.73 | 7.92 | 5.96 |
| Poor rotamers (%) | 1.59 | 0.87 | 6.61 | 1.12 |
| Ramachandran plot | | | | |
| Favored (%) | 89.09 | 88.20 | 89.66 | 90.11 |
| Allowed (%) | 10.91 | 11.80 | 10.34 | 9.89 |
| Disallowed (%) | 0.00 | 0.00 | 0.00 | 0.00 |

**Table S1. Cryo-EM data collection, refinement and validation statistics**
